# Clinician Discussion of Family Cancer Risk and Use of Genetic Testing Among US Adults With a Family History of Cancer

**DOI:** 10.5888/pcd23.260179

**Published:** 2026-10-15

**Authors:** Abe E. Sahmoun

**Affiliations:** 1 Department of Internal Medicine, University of North Dakota School of Medicine and Health Sciences, Fargo, North Dakota

## Abstract

**Introduction:**

Family history plays a key role in assessing inherited cancer risk, but genetic testing is still underused at the population level. The role of clinician–patient discussion in this gap is not well characterized. This study aimed to evaluate the association between clinician discussion of family cancer risk and use of genetic testing among US adults with a first-degree family history of cancer.

**Methods:**

This cross-sectional study used data from the 2023 National Health Interview Survey. The exposure was self-reported clinician discussion of family cancer risk, and the outcome was self-reported receipt of genetic testing for cancer risk. Survey weights were applied to generate nationally representative estimates. Propensity scores were used to match respondents with and without clinician discussion. Survey-weighted logistic regression models estimated associations.

**Results:**

Among 9,476 respondents (representing approximately 72.6 million US adults), 50% reported clinician discussion, and 7.8% reported genetic testing. Testing was more common among those reporting discussion (12.9%) than those who did not (2.7%) (*P* < .001). In fully adjusted analyses, clinician discussion was associated with higher odds of testing (adjusted odds ratio, 5.35; 95% CI, 4.23–6.76). Older age, female sex, higher education, and personal cancer history were also associated with testing.

**Conclusion:**

Clinician discussion of family cancer risk was strongly associated with use of genetic testing; however, overall testing rates were low. Improving routine clinician engagement may increase use of genetic services and support cancer prevention.

**Summary**

**What is already known on this topic?**

Family history is central to identifying risk of hereditary cancer, yet use of genetic testing is low in the US.

**What is added by this report?**

Clinician discussion of family cancer risk is strongly associated with use of genetic testing in a nationally representative sample.

**What are the implications for public health practice?**

Promoting routine clinician discussion may help to identify people at high risk of hereditary cancer and increase engagement with genetic services.

## Introduction

Cancer is a leading cause of death among people aged younger than 85 years in the US (1). Family history is a core component of hereditary cancer risk assessment and guides referral to genetic counseling and germline testing (2,3). Despite expanded availability of multigene panel testing and advances in precision prevention (4–6), population-level use of hereditary cancer testing remains low.

Approximately one-third of US adults report at least 1 first-degree relative with cancer (7). These people may benefit from risk-informed surveillance, cascade testing, and preventive interventions (8). However, many have never discussed their family cancer history with a clinician (9), highlighting persistent gaps between guideline recommendations and clinical implementation (10,11).

Prior research has focused largely on people diagnosed with cancer or people referred for genetic services, limiting generalizability (12). Population-based studies have not isolated clinician discussion as a primary exposure linking family history to testing. Clinician discussion may represent a potentially modifiable health care interaction that facilitates referral, genetic counseling, and testing.

The objective of this study was to describe the association between clinician discussion of family cancer risk and use of genetic testing in a nationally representative sample of US adults with a first-degree family history of cancer and identify population-level correlates of testing.

## Methods

### Study design

This cross-sectional analysis used data from the 2023 National Health Interview Survey (NHIS), an annual, nationally representative household survey of the US civilian, noninstitutionalized population administered by the National Center for Health Statistics (13). The survey uses a multistage, stratified, cluster probability design. The 2023 NHIS sample adult response rate was 47%. This study adhered to STROBE (Strengthening the Reporting of Observational Studies in Epidemiology) guidelines for cross-sectional research and was approved by the University of North Dakota Institutional Review Board (IRB0006920; October 31, 2025).

### Study population

Adults aged 18 years or older who completed the NHIS were eligible. Respondents without a family cancer history or with missing data on exposure, outcome, or covariates, as well as those reporting significant difficulty remembering or concentrating, were excluded (Figure). Overall missingness ranged from 0.02% to 6.5%; therefore, complete-case analysis without imputation was used.

**Figure:**
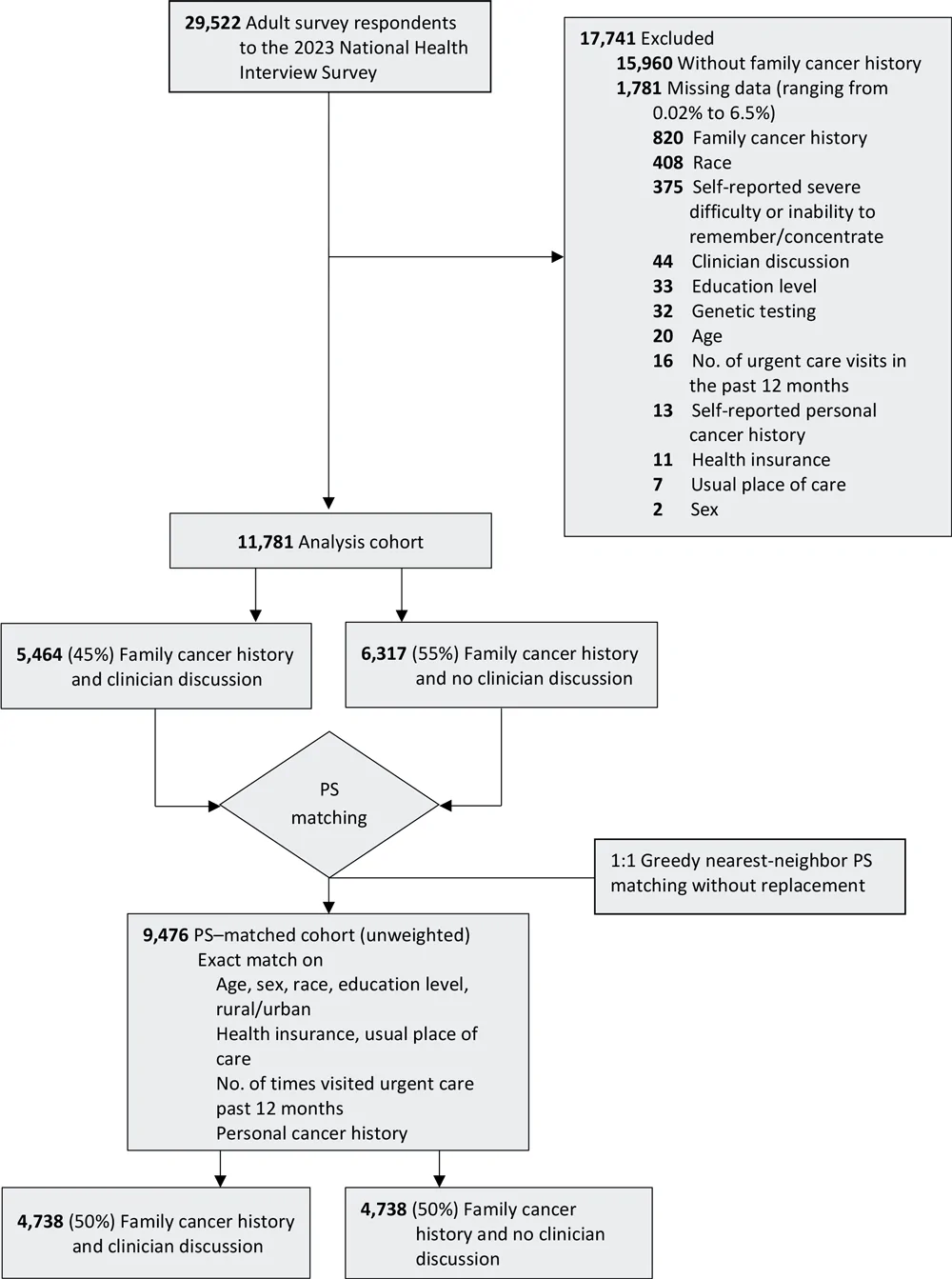
Flow diagram for a study on clinician discussion of family cancer risk and use of genetic testing among US adults with a family history of cancer. The PS matched cohort (unweighted n = 9,476) represented 72.6 million US adults. Percentages account for the complex survey design by incorporating strata, primary sampling units, and sampling weights. Abbreviation: PS, propensity score. Data source: 2023 National Health Information Survey (13).

### Family cancer history

Family cancer history was assessed by self-report of cancer among first-degree biological relatives (parents, siblings, or children), excluding skin cancer. Self-reported personal cancer history was assessed using the question, “Have you ever been told by a doctor or other health professional that you had cancer or a malignancy of any kind?” Responses were dichotomized as yes or no.

### Exposure, outcome, and covariates

Clinician discussion was defined as self-report of ever discussing family cancer risk with a doctor or other health professional, based on standardized NHIS questionnaire items (yes or no). The outcome was self-reported receipt of genetic testing for cancer risk (yes or no). This measure reflects respondent perception and may include misclassification (eg, tumor-based or direct-to-consumer testing). Both exposure and outcome were assessed as lifetime measures. Covariates were selected a priori and included age, sex, race and ethnicity, educational attainment, rural residence (vs urban), health insurance status (has or does not have), usual source of care (yes or no), health care utilization (≥2 health care visits in past 12 months), and personal history of cancer (yes or no). Rural residence was included because geographic location may influence access to specialty care, including genetic counseling and hereditary cancer testing services and because of differences in health care infrastructure and travel burden.

### Statistical analysis

All analyses incorporated NHIS sampling weights, strata, and primary sampling units to account for the complex survey design and to generate nationally representative estimates. Propensity score matching was used to improve covariate balance and comparability between respondents with and without clinician discussion, thereby reducing confounding by measured characteristics (14,15).

Propensity scores were estimated for reporting clinician discussion of family cancer risk using multivariable logistic regression without incorporation of survey weights, consistent with common practice for improving covariate balance (16,17). Matching on age, sex, race and ethnicity, educational attainment, rural residence, health insurance status, usual source of care, health care utilization, and personal history of cancer was conducted on the unweighted sample. A 1:1 nearest-neighbor matching without replacement was performed, and a caliper width of 0.15 of the standard deviation of the logit of the propensity score was used. Covariate balance was assessed using standardized mean differences.

Primary analyses were conducted in the matched sample using survey weights, strata, and primary sampling units. Survey-weighted multivariable logistic regression models were used to estimate the association between clinician discussion and use of genetic testing. Three models were fit: 1) unadjusted, 2) adjusted for sociodemographic characteristics (age, sex, race and ethnicity, educational attainment, rural residence, and health insurance), and 3) fully adjusted, additionally including usual source of care, health care utilization, and personal cancer history. Regression adjustment was retained after matching to account for any residual covariate imbalance. Standard errors were estimated by using survey design variables. All tests were 2-sided, and significance was defined as *P* < .05. Analyses were conducted using SAS version 9.4 (SAS Institute Inc).

Because exposure and outcome were assessed as lifetime measures in a cross-sectional design, temporal ordering could not be established, and findings are interpreted as associations rather than causal effects.

## Results

The analytic sample included 9,476 adults with a first-degree family history of cancer (mean age, 55.5 y), representing approximately 72.6 million US adults. Overall, 50% reported clinician discussion (Table 1).

**Table 1:** Characteristics of US Adults With a First-Degree Family History of Cancer, by Clinician Discussion Status, National Health Interview Survey, 2023

Characteristic	Weighted % (SE)^a^			*P* value^b^
	Total (N = 9,476)	Discussion (n = 4,738)	No discussion (n = 4,738)	
Age, mean (SE), y	55.5 (0.2)	56.3 (0.3)	54.7 (0.3)	<.001
Female sex	55.9 (0.6)	56.0 (0.8)	55.9 (0.9)	.92
Race				
Asian only	4.3 (0.3)	4.2 (0.4)	4.5 (0.4)	.35
Black or African American only	10.1 (0.5)	9.5 (0.6)	10.7 (0.7)	
White only	83.2 (0.7)	84.0 (0.8)	82.3 (0.8)	
Other or multiracial^c^	2.4 (0.2)	2.3 (0.3)	2.5 (0.3)	
Bachelor’s degree or higher	38.5 (0.7)	38.4 (0.9)	38.6 (0.9)	.80
Rural residence	14.5 (0.5)	14.6 (0.7)	14.3 (0.7)	.72
Health insurance	97.5 (0.2)	97.4 (0.3)	97.5 (0.3)	.82
Usual source of care	95.7 (0.3)	95.8 (0.3)	95.6 (0.4)	.74
≥2 Health care visits in past 12 months	14.9 (0.5)	15.7 (0.7)	14.1 (0.6)	.07
Personal history of cancer	15.8 (0.4)	17.3 (0.6)	14.3 (0.6)	<.001

a Values are weighted percentage (SE) unless otherwise indicated.

b Determined by Rao–Scott χ^2^ test.

c Includes American Indian or Alaska Native only, American Indian or Alaska Native and any other group, and other single and multiple races.

Overall, 7.8% reported undergoing genetic testing for hereditary cancer. Testing prevalence was substantially higher among respondents reporting clinician discussion than among those without discussion (12.9% vs 2.7%; *P* < .001).

In propensity score–matched analyses, clinician discussion was strongly associated with genetic testing. Respondents reporting discussion had more than 5-fold higher odds of testing than those who did not (adjusted odds ratio [AOR] = 5.35; 95% CI, 4.23–6.76) (Table 2).

**Table 2:** Association Between Clinician Discussion of Family Cancer Risk and Genetic Testing Uptake, National Health Interview Survey, 2023

Exposure	Unadjusted OR (95% CI) [*P* value]	Adjusted OR (95% CI)^a^ [*P* value]	Fully adjusted OR (95% CI)^b^ [*P* value]
No discussion	1 [Reference]	1 [Reference]	1 [Reference]
Discussion	5.23 (4.14–6.60) [<.001]	5.42 (4.29–6.85) [<.001]	5.35 (4.23–6.76) [<.001]

Abbreviation: OR, odds ratio.

a Adjusted for age, sex, race and ethnicity, educational attainment, rural residence, and health insurance.

b Additionally adjusted for usual source of care, health care utilization, and personal history of cancer.

Older age, female sex, higher education, and personal cancer history were independently associated with testing. Race and ethnicity, rural residence, health insurance, usual source of care, and health care utilization were not significant.

In stratified analyses, clinician discussion remained strongly associated with testing among respondents with and without a personal history of cancer. Among respondents without a personal history of cancer, clinician discussion was associated with substantially higher odds of testing (AOR = 5.54; 95% CI, 4.21–7.30). A similar association was observed among respondents with a personal cancer history (AOR = 5.00; 95% CI, 3.29–7.61), indicating that the primary findings were not driven solely by testing related to cancer diagnosis, treatment, or surveillance. Genetic testing was approximately 10 percentage points more common among respondents reporting clinician discussion.

## Discussion

In this nationally representative study, clinician discussion of family cancer risk was strongly associated with use of genetic testing. This association persisted after adjustment and among people without a personal cancer history, suggesting relevance for preventive care. The magnitude of the observed association suggests that clinician discussion may function less as an isolated exposure and more as a marker of underlying access to care, health system engagement, and risk awareness.

Prior population-based studies primarily described the prevalence of hereditary cancer testing or testing patterns within populations referred to oncology services (18,19). The findings of this study extend this literature by demonstrating, in a nationally representative sample of US adults with a first-degree family history of cancer, that clinician discussion of family cancer risk is strongly associated with use of genetic testing independent of sociodemographic and health care access factors. The persistence of this association among people without a personal history of cancer further highlights the potential relevance of clinician engagement for preventive care and risk assessment beyond health care settings designed specifically for the diagnosis, treatment, and ongoing care of people with cancer.

Because this study assessed clinician discussion and genetic testing as lifetime events, the direction of the association cannot be determined. Clinician discussion may precede testing, but genetic testing itself or related clinical encounters may also have prompted subsequent discussion. More broadly, both may reflect underlying health care engagement, access, or risk perception rather than a direct causal pathway. Accordingly, these results should be interpreted as associations rather than evidence of causation.

These findings highlight a population-level gap between identification of cancer risk and engagement with genetic services. Clinician discussion may serve as an entry point to referral, genetic counseling, and cascade testing.

Improving systematic collection of family cancer history and facilitating clinician discussion may increase use of genetic testing and support early identification of people at high risk of hereditary cancer. Ensuring equitable clinician engagement is critical to prevent widening disparities in access to genetic services (4,20).

### Limitations and strengths

This study has several limitations. Measures were self-reported and subject to recall bias and misclassification. NHIS does not distinguish germline testing from tumor testing, nor does it capture detailed family history (21,22), potentially limiting risk stratification. Residual confounding by health-seeking behavior and genetic literacy is possible. Reverse causation is also possible because clinician discussion may have occurred after genetic testing or other cancer-related clinical events rather than before testing. Misclassification of testing is likely nondifferential and may bias results toward the null. Strengths include a large, nationally representative sample and rigorous analytic methods.

### Conclusion

Enhancing clinician discussion of family cancer history may represent a scalable strategy to increase use of genetic testing and improve population-level cancer prevention.
